# Ichthyofauna in a highly urbanised area (Kitakyushu metropolitan area, Fukuoka, Japan)

**DOI:** 10.3897/BDJ.13.e155035

**Published:** 2025-11-28

**Authors:** Rei Itsukushima, Keigo Otsu, Soma Matsushima, Hironari Miyahara

**Affiliations:** 1 Kyushu Institute of Technology, Kitakyushu, Japan Kyushu Institute of Technology Kitakyushu Japan; 2 Kyushu University, Fukuoka, Japan Kyushu University Fukuoka Japan

**Keywords:** channel degradation, fish fauna, habitat loss, invasive species, urbanisation

## Abstract

**Background:**

Urbanisation has caused significant changes in material cycles, alterations in river flow rates and depths and modifications to habitats for aquatic organisms. These changes have led to disruptions in the movement and dispersal of aquatic species, as well as shifts in biodiversity. The loss of habitats due to urbanisation has resulted in a decline in species diversity across a variety of taxonomic groups. Aquatic organisms, in particular, are adversely affected by habitat loss caused by river channel modifications and the invasion of non-native species. It is generally reported that, in regions where invasive species have established and native species have declined, fish fauna tend to homogenise, leading to a substantial reduction in diversity. However, the impact of urbanisation on fish fauna varies depending on local ecosystems, the degree of urbanisation and the time elapsed since urban development. Therefore, it is crucial to gather region-specific information on how fish fauna have been altered by urbanisation. The study area in this research, the Kitakyushu metropolitan area, is one of Japan's oldest heavy industrial zones and many of its rivers have been affected by channel straightening and concrete lining.

**New information:**

A fish survey was conducted at 100 locations within the Kitakyushu metropolitan area during the summer period (from 16 July to 4 October 2024), focusing on river sections modified by channel straightening and concrete lining due to urbanisation. The survey resulted in collecting 30 genera and 32 species from nine orders and 19 families, for a total of 9,253 individuals. Amongst the species collected, rare species, such as the Bitterling, *Liobagrus reini* and *Misgurnus anguillicaudatus*, were also found (The Japanese Red List). Although the number of individuals was limited, these native rare species were found in inhabited rivers significantly altered by human activity. Additionally, invasive species including *Lepomis macrochirus*, *Micropterus salmoides* (Lacepède, 1802) and *Channa argus* (Cantor, 1842) were recorded. Despite accounting for only 0.5% of the total fish population, the highly predatory nature of these invasive species warrants proactive management, including the enhancement of impounded water environments through structures such as weirs, to mitigate their potential ecological impact.

## Introduction

Urban populations continue to grow and the importance of cities is increasing when considering the global environment. In recent years, the significance of ecosystem services provided by urban rivers has been recognised (Chien and Saito 2021) and the health of urban rivers is now seen as a critical element for achieving sustainable urban development (Guimarães et al. 2021). For the natural restoration of urban rivers, a systematic and quantitative understanding of the impacts of urbanisation on rivers is essential, yet scientific knowledge in this area remains insufficient. In Japan, due to the need to address increased flood flows resulting from urbanisation and the constraints of available land, most rivers in urban areas have been primarily designed for flood control, often without sufficient consideration for environmental factors. Urban waterbodies, particularly urban streams, possess the potential to offer a diverse array of ecosystem services, ranging from habitat and energy provision to pollutant removal, improved amenities and recreational opportunities. In densely developed urban areas, they can serve as critical focal points for re-establishing connections between humans and the natural environment (Lundy and Wade 2011). There is growing recognition within the country of the importance of the ecosystem services provided by urban rivers and momentum for natural restoration is steadily increasing (Itsukushima and Ohtsuki 2021).

Habitat loss, pollution, contamination and the reduction of resources for basal species —thereby affecting the entire food chain — due to urbanisation has led to a decline in species diversity across various taxonomic groups (Blair 1999, [Bibr B12260881][Bibr B12260854]). Amongst these, aquatic organisms are particularly affected by the loss of habitats due to river modifications and the invasion of non-native species as urbanisation progresses (Pauchard et al. 2006, Engman and Ramírez 2012). Fish, as top consumers in river ecosystems, are amongst the most sensitive taxa to urbanisation (Kollaus et al. 2014). In general, regions where invasive species have established themselves and native species have declined show evidence of homogenisation of fish communities, leading to significant reductions in biodiversity (Marchetti et al. 2006, Olden et al. 2006). In contrast, despite 85% of the watershed being urbanised and the majority of the Tsurumi River, which flows through Tokyo and Kanagawa, being artificially modified, rare fish species have been observed in certain areas. For example, the *Tanakia lanceolata* (Temminck & Schlegel, 1846), which was previously believed to be extinct in the study area, has been found in the low-velocity zones near tributary confluences. Additionally, in areas where riparian forests have been minimally preserved, even in sections of the river that are lined with concrete, the presence of the endangered *Lefua echigonia* (Jordan and Richardson, 1907) has been confirmed (Itsukushima and Maruoka 2022, Itsukushima et al. 2024). Thus, it has been suggested that, even in urban watersheds with degraded environmental conditions, substantial improvements in biodiversity can be achieved through natural restoration. Furthermore, although urban rivers often suffer from environmental degradation and insufficient data on their biota, the importance of conducting thorough surveys of the biological communities in urban areas for effective environmental restoration has been emphasised.

Although interest in the biodiversity of urban rivers is increasing, there is still a lack of sufficient data and current knowledge remains inadequate to effectively support the restoration and conservation of urban river environments. The target area of this study, the Kitakyushu metropolitan area, is one of Japan's oldest industrial zones, where heavy chemical industries, particularly the steel industry, have been prominent since the early 20^th^ century. As a result of urbanisation, many of the region's rivers have been transformed into concrete-lined channels, leading to the anticipated degradation of river ecosystems (Suppl. material 1). However, due to the large number of small and medium-sized rivers, monitoring by government agencies and local authorities has been limited and there is a lack of comprehensive information on the regional biota. This paper reports the findings of a survey on the fish fauna of the Kitakyushu metropolitan area, where such data are scarce. The study aims to provide critical foundational knowledge for the environmental conservation and natural restoration of small and medium-sized urban rivers, contributing to a better understanding and management of these ecosystems in the face of urban pressures.

## Sampling methods

### Study extent

**Figure 1.**
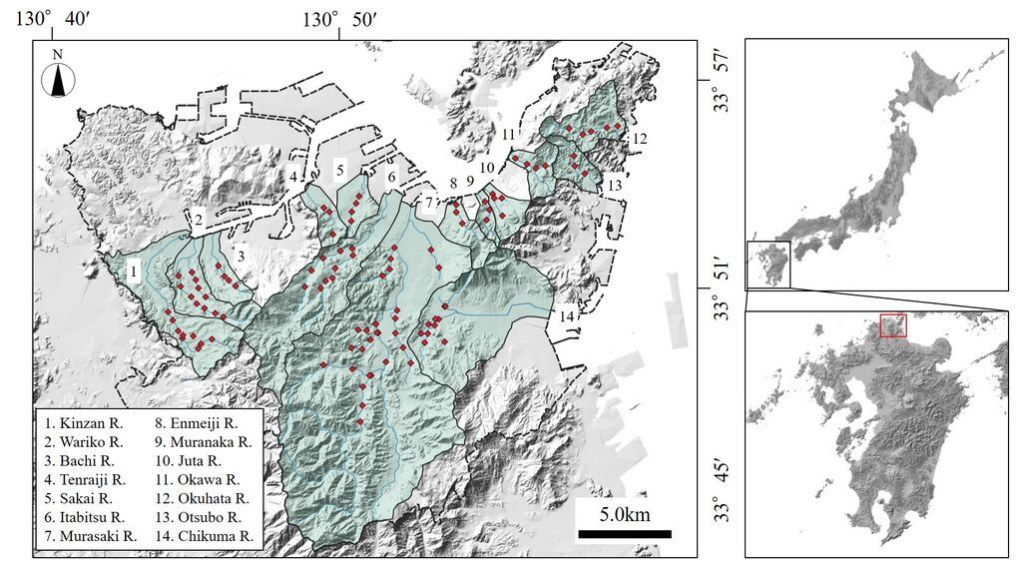
Location of the study sites. The study focused on 100 locations across 14 river systems.

This study was conducted at 100 sites across 14 river basins flowing through the Kitakyushu metropolitan area in Japan (Fig. 1). The selected rivers ranged in watershed area from 2.1 km² (Muranaka River) to 113 km² (Murasakigawa River), representing small and medium-sized rivers. Kitakyushu City, the core of the Kitakyushu Industrial Zone, is one of Japan’s major industrial regions and a hub for heavy and chemical industries, particularly iron and steel production. With industrialisation, the population became concentrated and although it has declined in recent years, the City had a population of 939,000 in 2020. Many of the small and medium-sized rivers in the area have been canalised with concrete, leading to the degradation of river ecosystems in numerous locations. However, there is a lack of comprehensive biological data for the broader region of Kitakyushu. Thus, fish surveys were conducted at 100 sites, primarily focusing on smaller rivers where data are scarce. In this study, a total of 9,253 individuals were recorded, with species identified both on-site and in the laboratory according to the identification keys of Kawanabe and Mizuno (1989) and Seno (2007). The fish surveys were carried out during the summer period (from 16 July to 4 October 2024). The average daily temperature on the survey days was 26.5°C, according to data from the Japan Meteorological Agency's Yahata observatory.

### Sampling description

The fish survey was conducted using a pulsed DC Smith-Root Model 12-A p DC backpack electrofisher (Smith-Root Inc., Vancouver, WA, U.S.A.) at each habitat type (rapid, run, pool, glide, slack and backwater) across 100 survey stations. The length of each survey section was one river reach, approximately 10 times the width of the river channel. The electrofisher was set to an output voltage of 120 V and operated in the standard pulse mode, which delivers continuous pulsed waveforms at regular intervals (frequency: 60 Hz). The duration of use ranged from 30 to 90 minutes, depending on the size of the river channel and the number of habitats. We employed a backpack electrofisher due to its high capture efficiency through visual detection and netting, as it temporarily immobilises fish and induces surfacing. Moreover, since the fish recover within a short period and can be released back into the habitat, this method allows for the assessment of population size and diversity, while minimising stress on the fish. The scientific names of the taxa, their authorship and year of publication and original descriptions followed Fricke et al. (2025a) and Fricke et al. (2025b). In addition, to compare species diversity and the number of individuals between sites with and without the presence of non-native species, the Wilcoxon rank-sum test was conducted using the statistical software R (version 4.5.1). The significance level was set at 5% (p < 0.05).

## Geographic coverage

### Description

Surveys were conducted at 100 sites in the 14 river systems located in the Kitakyushu metropolitan area during summer, focusing on river channels that have been straightened or converted to concrete channels due to urbanisation.

### Coordinates

33.78448 and 33.92905 Latitude; 130.73471 and 130.99965 Longitude.

## Taxonomic coverage

### Description

**Characteristics of the fish survey results**

As a result of the surveys, nine orders, 19 families, 30 genera and 32 species were identified, with 9,253 individuals being collected from the 100 stations. The highest species richness was recorded at sites belonging to the Murasaki River and Shii River, with 14 species collected. The greatest number of individuals was recorded at a site belonging to the Itabitsu River, with 548 individuals being collected. By contrast, fish were not confirmed in the site belonging to the Okuhata R. The highest number of individuals found was 3,146 of *Nipponocypris temminckii* (Temminck et Schlegel, 1846), which appeared in 76 stations (Table 1).

**Taxonomic coverage**

**Figure 2.**
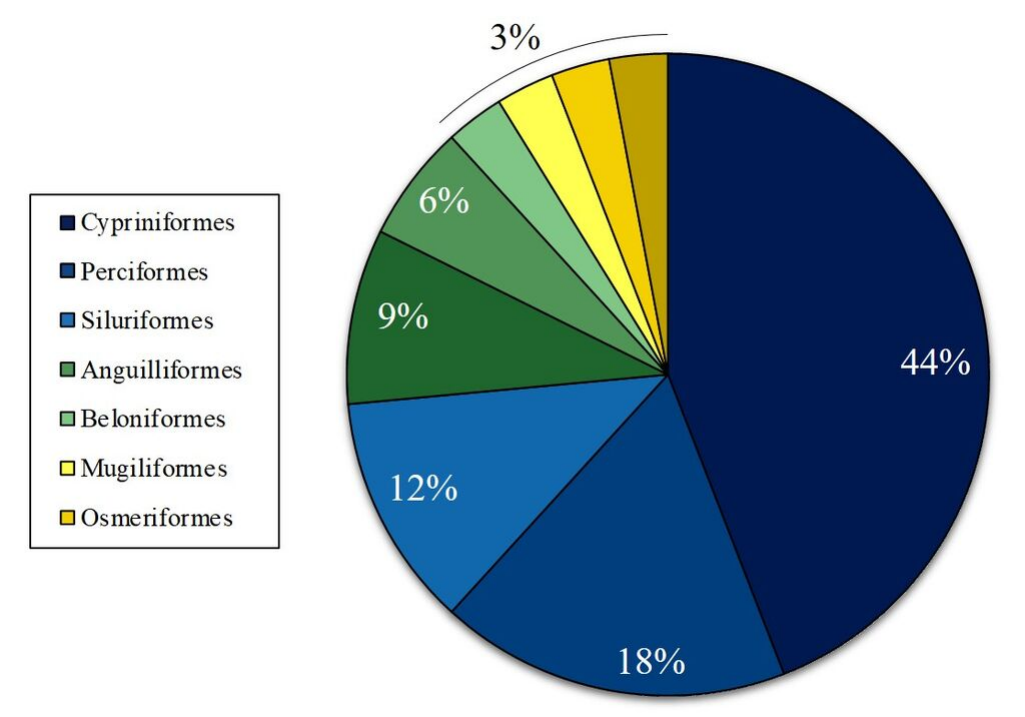
Taxonomic coverage (by order).

**Figure 3.**
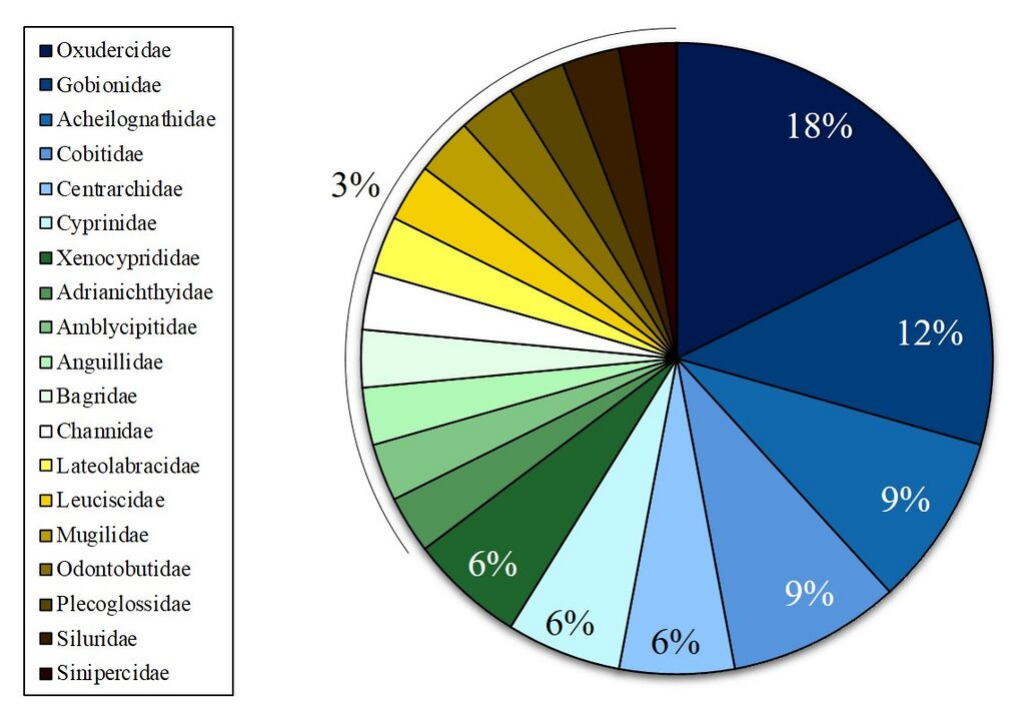
Taxonomic coverage (by family).

The orders were Cypriniformes (15 species), Gobiiformes (6 speices), Perciformes (4 species), Siluriformes (3 species), Centrarchiformes (2 species), Anguilliformes (1 species), Beloniformes (1 species), Mugiliformes (1 species) and Osmeriformes (1 species) (Fig. 2). The families were Oxudercidae (6 species), Gobionidae (4 species), Acheilognathidae (3 species), Cobitidae (3 species), Centrarchidae (2 species), Cyprinidae (2 species), Xenocyprididae (2 species), Adrianichthyidae (1 species), Amblycipitidae (1 species), Anguillidae (1 species), Bagridae (1 species), Channidae (1 species), Lateolabracidae (1 species), Leuciscidae (1 species), Mugilidae (1 species), Odontobutidae (1 species), Plecoglossidae (1 species), Siluridae (1 species) and Sinipercidae (1 species) (Fig. 3).

According to the Red Data Book published by the Fukuoka Prefecture in 2014, *Anguilla japonica* Temminck & Schlegel 1846 was determined as EN (Endangered). *Tanakia lanceolata* (Temminck & Schlegel, 1846), *Misgurnus anguillicaudatus* (Cantor, 1842) and *Liobagrus reinii* Hilgendorf 1878 were determined as VU (Vulnerable). *Plecoglossus altivelis* (Temminck & Schlegel, 1846), *Acheilognathus rhombeus* (Temminck & Schlegel, 1846), *Oryzias latipes* (Temminck & Schlegel, 1846), *Cobitis matsubarae* Okada & Ikeda 1939 and *Tanakia limbata* (Temminck & Schlegel, 1846) were determined as NT (Near Threatened). In the Ministry of the Environment's Red Data Book (2020) for Japan, *Plecoglossus altivelis* and *Acheilognathus rhombeus* are not listed amongst the species mentioned above. On the other hand, *Coreoperca kawamebari* (Temminck & Schlegel, 1843) is listed as EN (Endangered).

## Usage licence

### Usage licence

Other

### IP rights notes

This work is licensed under a Creative Commons Attribution (CC-BY 4.0) Licence.

## Data resources

### Data package title

A database of fish fauna in Kitakyushu metropolitan area

### Resource link

https://doi.org/10.15468/tb9cvk

### Alternative identifiers

https://ipt.pensoft.net/resource?r=database_fish_fauna_kitakyushu

### Number of data sets

1

### Data set 1.

#### Data set name

A database of fish fauna in Kitakyushu metropolitan area

#### Download URL

https://ipt.pensoft.net/archive.do?r=database_fish_fauna_kitakyushu&v=1.8

#### Description

Surveys were conducted at 100 sites in the 14 river systems located in the Kitakyushu metropolitan area, focusing on river channels that have been straightened or converted to concrete channels due to urbanisation. As a result of this investigation, 30 genera, 32 species and 9,253 individuals were collected (Itsukushima 2025).

**Data set 1.** 

Column label	Column description
occurrenceID	An identifier for the Occurrence.
basisOfRecord	The specific nature of the data record.
samplingProtocol	The names of, references to, or descriptions of the methods or protocols used during an Event.
eventDate	The date-time or interval during which an Event occurred.
scientificName	The full scientific name.
scientificNameAuthorship	The authorship information for the scientificName formatted according to the conventions of the applicable nomenclaturalCode.
kingdom	The full scientific name of the kingdom in which the taxon is classified.
phylum	The full scientific name of the phylum or division in which the taxon is classified.
class	The full scientific name of the class in which the taxon is classified.
order	The full scientific name of the order in which the taxon is classified.
family	The full scientific name of the family in which the taxon is classified.
taxonRank	The taxonomic rank of the most specific name in the scientificName as it appears in the original record.
identifiedBy	A list (concatenated and separated) of names of people, groups or organisations who assigned the Taxon to the subject.
recordedBy	A list (concatenated and separated) of the globally unique identifier for the person, people, groups or organisations responsible for recording the original Occurrence.
decimalLatitude	The geographic latitude (in decimal degrees, using the spatial reference system given in geodeticDatum) of the geographic centre of a Location.
decimalLongitude	The geographic longitude (in decimal degrees, using the spatial reference system given in geodeticDatum) of the geographic centre of a Location.
coordinateUncertaintyInMetres	The horizontal distance (in metres) from the given decimalLatitude and decimalLongitude describing the smallest circle containing the whole of the Location.
geodeticDatum	The ellipsoid, geodetic datum or spatial reference system (SRS) upon which the geographic coordinates given in decimalLatitude and decimalLongitude are based.
countryCode	The standard code for the country in which the Location occurs. Recommended best practice is to use ISO 3166-1-alpha-2 country codes.
individualCount	The number of individuals represented present at the time of the Occurrence.
occurrenceStatus	A statement about the presence or absence of a Taxon at a Location.
catalogNumber	A list (concatenated and separated) of previous or alternative fully qualified catalogue numbers or other human-used identifiers for the same Occurrence, whether in the current or any other dataset or collection.
language	A language of the resource. Recommended best practice is to use a controlled vocabulary, such as RFC 4646 [RFC4646].
country	The name of the country or major administrative unit in which the Location occurs. Recommended best practice is to use a controlled vocabulary, such as the Getty Thesaurus of Geographic Names.
stateProvince	The name of the next smallest administrative region than country (state, province, canton, department, region etc.) in which the Location occurs.
municipality	The full, unabbreviated name of the next smallest administrative region than county (city, municipality etc.) in which the Location occurs. Do not use this term for a nearby named place that does not contain the actual location.
waterBody	The name of the waterbody in which the dcterms:Location occur.
modified	The most recent date-time on which the resource was changed. For Darwin Core, recommended best practice is to use an encoding scheme, such as ISO 8601:2004(E).
year	The four-digit year in which the Event occurred, according to the Common Era Calendar.
month	The ordinal month in which the Event occurred.
day	The integer day of the month on which the Event occurred.
informationWithheld	Additional information that exists, but that has not been shared in the given record.
establishmentMeans	Statement about whether a dwc:Organism has been introduced to a given place and time through the direct or indirect activity of modern humans.
dynamicProperties	A list of additional measurements, facts, characteristics or assertions about the record. Meant to provide a mechanism for structured content. Here we provided national protective status: "endangered," "near threatened" and "vulnerable".
genus	The full scientific name of the genus in which the taxon is classified.

## Additional information

As a result of this survey, 29 native species and three non-native species were confirmed at the surveyed sites. The average number of native fish species in the West Setouchi Ecological Region, to which the Kitakyushu metropolitan area belongs, is 34.5 species (Itsukushima 2019) and the findings of this survey were below that value. This outcome is likely because the rivers surveyed are highly urbanised, leading to significant habitat degradation. Furthermore, the rivers surveyed are relatively small in scale and the absence of alternative habitats following river modification may be a contributing factor to the decline in species.

The northern part of Kyushu, where the study area is located, is known to be the most species-rich region for bitterlings in Japan, with six species present (Onikura et al. 2006). In this survey, three species of the bitterlings were confirmed, with *Tanakia limbata* being the most abundant, with 56 individuals recorded. Although bitterlings are currently facing significant declines due to predation by non-native species, river modifications and field improvements (Negishi et al. 2008, Kitamura and Morosawa 2010), the results of this survey indicate that several species of bitterlings can still be found in urban rivers with degraded environments. This suggests that, with riverbed restoration and floodplain rehabilitation, there is potential for population recovery.

In addition, the *Anguilla japonica*, which is classified as an endangered species, was recorded at 15 sites, with a total of 51 individuals. This species was primarily observed in rivers that flow into relatively large tidal flats with optimal environmental conditions, such as the Okuhata River and Otsubo River. However, it was also confirmed in highly urbanised watersheds, such as the Sakai River and Tenraiji River, where the majority of the river bed is lined with concrete, indicating that the species can persist in highly altered environments. These findings underscore the species' capacity to adapt to diverse habitats and suggest that restoration efforts in urbanised areas could support its conservation. In particular, the Dokai Bay, into which both rivers flow, experienced significant water pollution during the period of rapid economic growth. A 1969 survey recorded a chemical oxygen demand (COD) of 48.4 mg/l and the Bay was even referred to as a "dead sea" due to the inability of coliform bacteria to survive (Matsuo 2008). Since then, improvements in wastewater infrastructure and factory discharge regulations have led to improvements in water quality (Yasuda et al. 2008). However, the presence of the *Anguilla japonica* in the concrete-lined channels of the rivers flowing into Dokai Bay in this study suggests that the recovery of coastal marine biodiversity due to water quality improvements has taken place. This also indicates that environmental restoration of the target rivers could significantly enhance the populations of migratory fish species that utilise both river and marine ecosystems.

**Figure 4.**
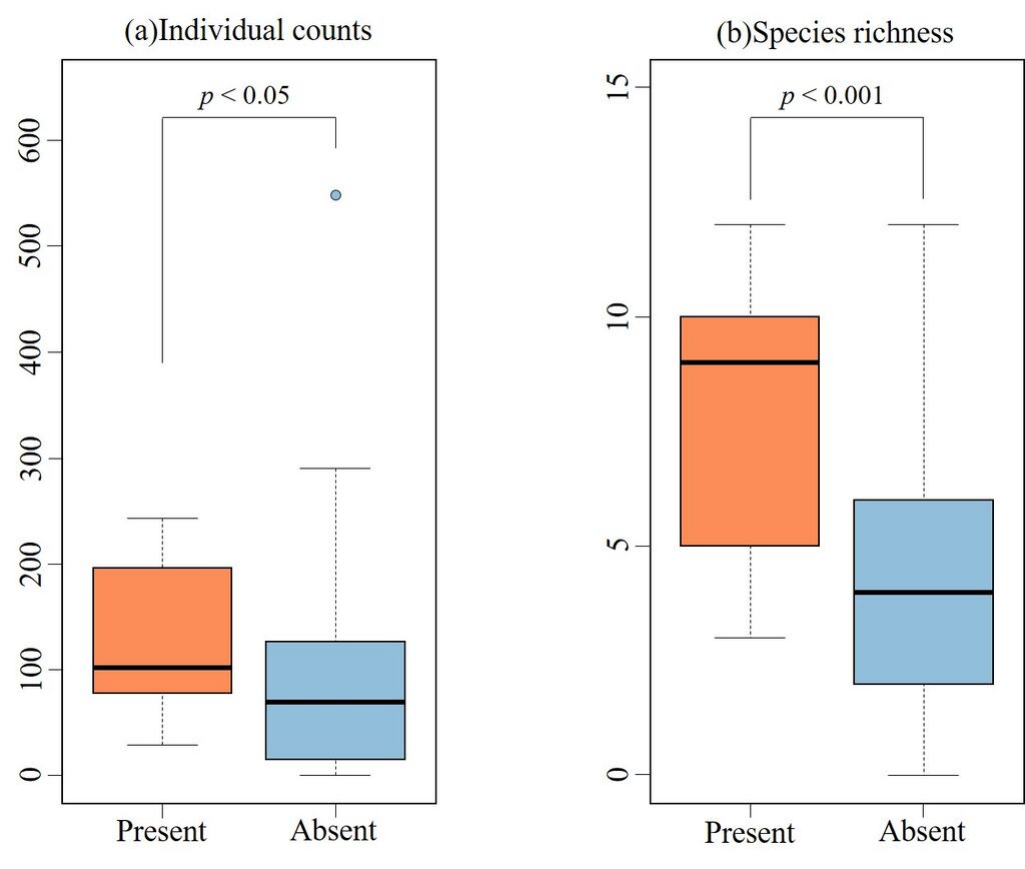
Differences in individual counts and species richness between occurrence and non-occurrence sites of invasive species. The number of sites where the target non-native species was present was 13, while it was absent at 87 sites. According to the results of the Wilcoxon rank-sum test, the *p*-values were 0.028 for the individual counts and 0.0008 for the species richness. **a** Individual counts; **b** Species richness.

In general, urbanisation has been shown to lead to the expansion of the distribution ranges of introduced species and the decline of native species, resulting in a decrease in species diversity and homogenisation in many regions (Gavioli et al. 2019). However, the results of this survey revealed only three non-native species – *Lepomis macrochirus*, *Micropterus salmoides* and *Channa argus* – with a total of 46 individuals (mean total length of the captured individuals was 128 ± 65 mm), representing less than 0.5% of the total catch. Despite being located in areas heavily influenced by anthropogenic factors, the presence of non-native fish was relatively low. This can be attributed to the fact that, at sites with significantly degraded environments, the concrete canalisation results in extremely shallow water depths that prevent the establishment of non-native fish populations (Itsukushima et al. 2024). In contrast, many of the captured non-native fish were observed in lentic habitats, such as oxbow lakes and impounded areas upstream of weirs, rather than in the main river channel. These stagnant environments are known to provide suitable habitats for predatory invasive species (Kawase et al. 2017). Their reproduction in such areas may lead to the replacement of native species through predation and result in biotic homogenisation (Hermoso et al. 2011). Since complete eradication of invasive species through continuous removal in river systems is generally difficult (Katano and Sakano 2010), it is essential to improve riverine environments, such as by integrating weir management, to create conditions less favourable for the establishment of highly predatory non-native fish. Additionally, the sites where non-native species were found tended to have relatively favourable environmental conditions, with high species diversity and large populations of native species, indicating that these areas do not support an environment dominated by non-native species (Fig. 4).

The results of this survey revealed that, despite significant environmental degradation due to land-use changes and river canalisation in urbanised small to medium-sized rivers, a total of 32 fish species were identified, including eight rare species. Additionally, although three predatory non-native species, known to threaten native ecosystems, were found in the study area, their populations were minimal in comparison to the overall fish community. This suggests that co-existence with native ecosystems may still be occurring. These findings indicate that, in the Kitakyushu metropolitan area, river ecosystem restoration has the potential to support the recovery of fish biodiversity.

## Supplementary Material

F2AA294B-64AA-53EE-A5DC-C9AB84615E13 10.3897/BDJ.13.e155035.suppl1 Supplementary material 1 Landscape photograph of the surveyed site Data type images Brief description Landscape photograph of the surveyed site with a concrete-lined channel. File: oo_1345545.pdf https://binary.pensoft.net/file/1345545 Itsukushima R

## Figures and Tables

**Table 1.** The occurrence of fish taxa in the Kitakyushu metropolitan area, Fukuoka, Japan. Rare species are indicated with * and invasive species with ** in the table.

Rank	Scientific Name	Number of occurrences
Order	Cypriniformes	
family	Cyprinidae	
species	*Cyprinus carpio* Linnaeus, 1758	41
species	*Carassius langsdorfii* Temminck & Schlegel, 1846	282
family	Acheilognathidae	
species	*Acheilognathus rhombeus* (Temminck & Schlegel, 1846)	1
species	*Tanakia lanceolata* (Temminck & Schlegel, 1846)*	9
species	*Tanakia limbata* (Temminck & Schlegel, 1846)*	56
family	Gobionidae	
species	*Squalidus gracilis* (Temminck & Schlegel 1846)	135
species	*Gnathopogon elongatus* (Temminck & Schlegel, 1846)	20
species	*Pseudogobio esocinus* (Temminck & Schlegel, 1846)	61
species	*Pungtungia herzi* Herzenstein, 1892	587
family	Xenocyprididae	
species	*Nipponocypris temminckii* (Temminck et Schlegel, 1846)	3146
species	*Zacco platypus* (Temminck et Schlegel, 1846)	1697
family	Leuciscidae	
species	*Rhynchocypris jouyi* (Jordan & Snyder, 1901)	60
family	Cobitidae	
species	Cobitis sp.	3
species	*Cobitis matsubarae* Okada & Ikeda, 1939*	22
species	*Misgurnus anguillicaudatus* (Cantor, 1842)*	55
Order	Gobiiformes	
family	Oxudercidae	
species	*Rhinogobius nagoyae* Jordan & Seale, 1906	858
species	*Rhinogobius* sp.	973
species	*Acanthogobius flavimanus* (Temminck & Schlegel, 1845)	11
species	*Rhinogobius similis* Gill 1859	163
species	*Tridentiger brevispinis* Katsuyama, Arai & Nakamura, 1972	100
species	*Gymnogobius urotaenia* (Hilgendorf, 1879)	20
Order	Centrarchiformes	
family	Centrarchidae	
species	*Lepomis macrochirus* Rafinesque, 1819**	19
species	*Micropterus salmoides* (Lacepède 1802)**	24
Order	Osmeriformes	
family	Plecoglossidae	
species	*Plecoglossus altivelis* (Temminck & Schlegel 1846)	35
Order	Perciformes	
family	Odontobutidae	
species	*Odontobutis obscura* (Temminck & Schlegel, 1845)	703
family	Lateolabracidae	
species	*Lateolabrax japonicus* (Cuvier, 1828)	1
family	Channidae	
species	*Channa argus* (Cantor, 1842)**	3
family	Sinipercidae	
species	*Coreoperca kawamebari* (Temminck & Schlegel, 1843)*	48
Order	Mugiliformes	
family	Mugilidae	
species	*Mugil cephalus* Linnaeus, 1758	3
Order	Siluriformes	
family	Bagridae	
species	*Tachysurus nudiceps* (Sauvage, 1883)	9
family	Amblycipitidae	
species	*Liobagrus reini* Hilgendorf, 1878*	1
family	Siluridae	
species	*Silurus asotus* Linnaeus, 1758	4
Order	Beloniformes	
family	Adrianichthyidae	
species	*Oryzias latipes* (Temminck & Schlegel, 1846)*	46
Order	Anguilliformes	
family	Anguillidae	
species	*Anguilla japonica* Temminck & Schlegel, 1846*	57
