# Supplementary figures and images for: Ichthyofauna in a highly urbanised area (Kitakyushu metropolitan area, Fukuoka, Japan)
Source: Biodivers Data J. 2025 Nov 28;13:e155035. doi: 10.3897/BDJ.13.e155035 (PMC12680944; doi:10.3897/BDJ.13.e155035)

Bachi R.

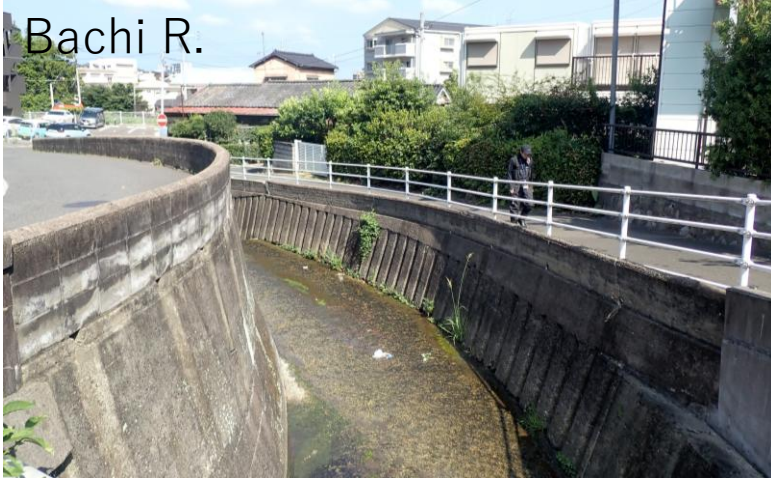

Okuhata R.

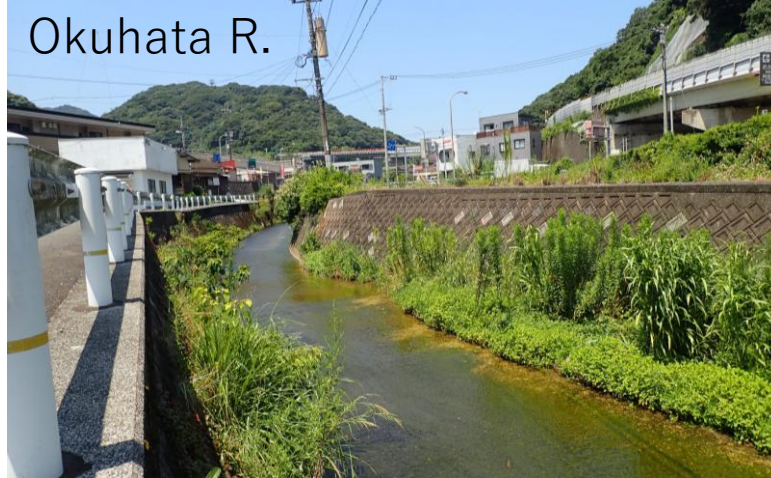

Okuhata R.

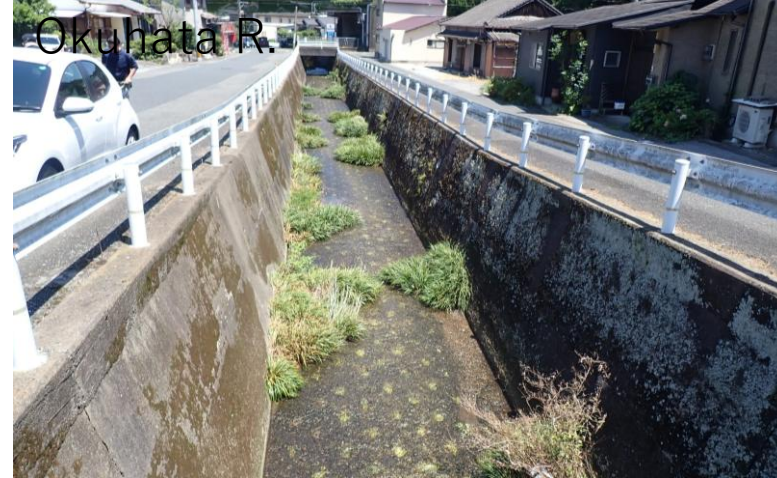

Miya R.

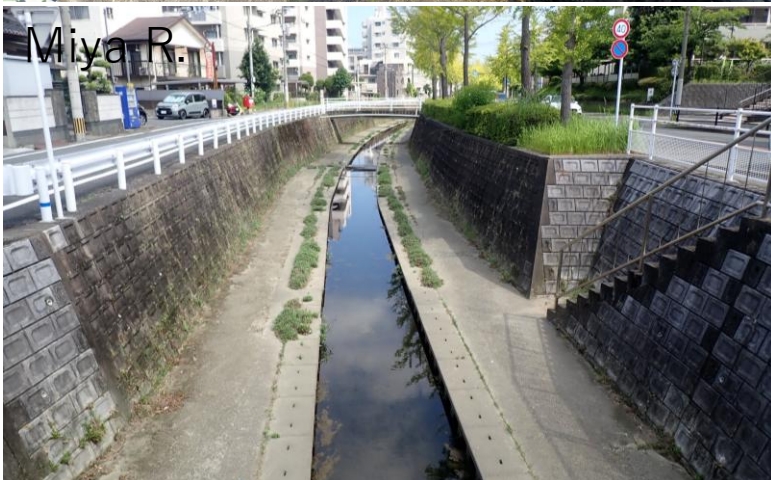

Okawa R.

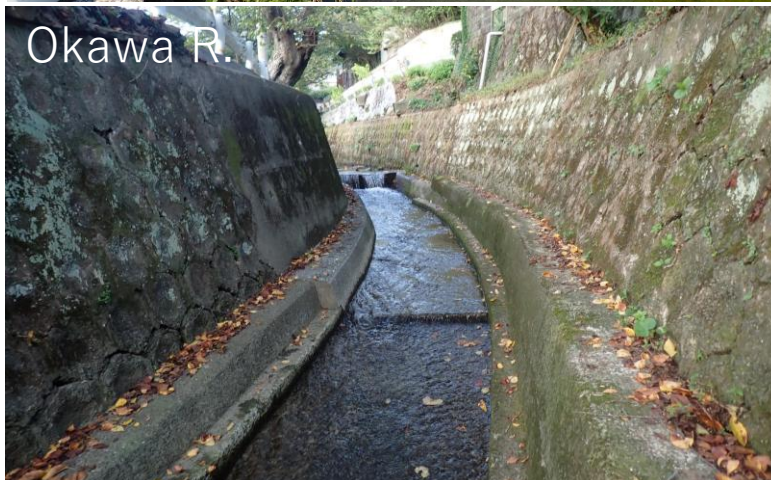

Miya R.

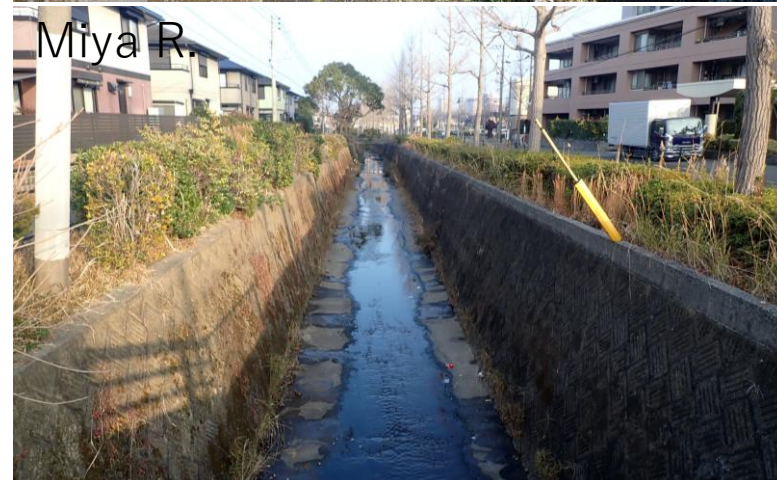

Jitta R.

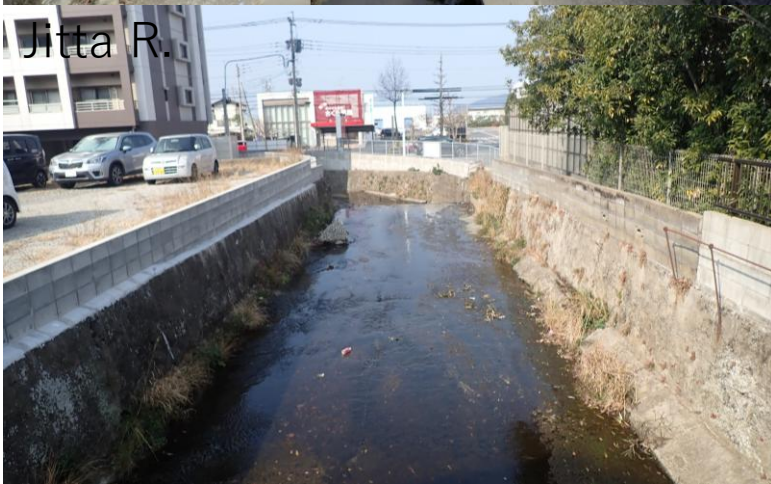

Muranaka R.

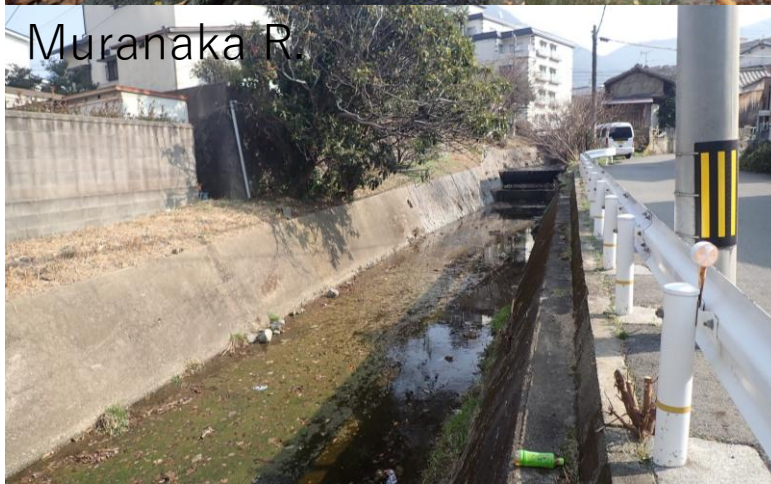

Muranaka R.

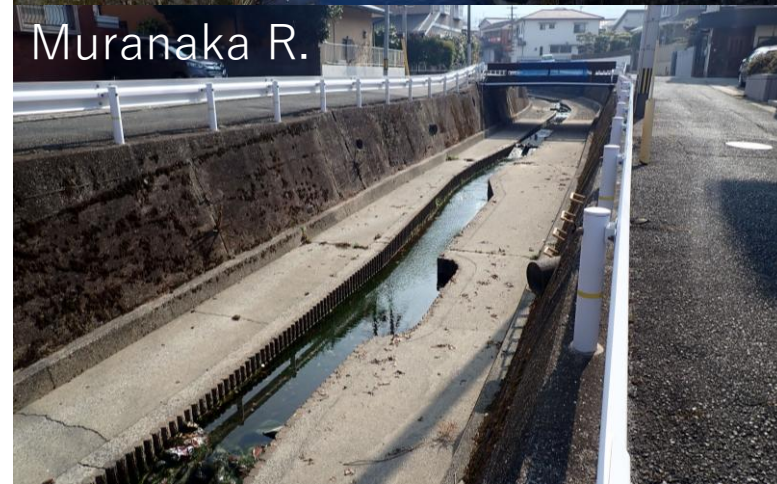

Supplement: Supplementary material 1 — Landscape photograph of the surveyed site [file bdj-13-e155035-s001.pdf]
